# Prevalence and risk factors of post-COVID-19 condition in adults and children at 6 and 12 months after hospital discharge: a prospective, cohort study in Moscow (StopCOVID)

**DOI:** 10.1186/s12916-022-02448-4

**Published:** 2022-07-06

**Authors:** Ekaterina Pazukhina, Margarita Andreeva, Ekaterina Spiridonova, Polina Bobkova, Anastasia Shikhaleva, Yasmin El-Taravi, Mikhail Rumyantsev, Aysylu Gamirova, Anastasiia Bairashevskaia, Polina Petrova, Dina Baimukhambetova, Maria Pikuza, Elina Abdeeva, Yulia Filippova, Salima Deunezhewa, Nikita Nekliudov, Polina Bugaeva, Nikolay Bulanov, Sergey Avdeev, Valentina Kapustina, Alla Guekht, Audrey DunnGalvin, Pasquale Comberiati, Diego G. Peroni, Christian Apfelbacher, Jon Genuneit, Luis Felipe Reyes, Caroline L. H. Brackel, Victor Fomin, Andrey A. Svistunov, Peter Timashev, Lyudmila Mazankova, Alexandra Miroshina, Elmira Samitova, Svetlana Borzakova, Elena Bondarenko, Anatoliy A. Korsunskiy, Gail Carson, Louise Sigfrid, Janet T. Scott, Matthew Greenhawt, Danilo Buonsenso, Malcolm G. Semple, John O. Warner, Piero Olliaro, Dale M. Needham, Petr Glybochko, Denis Butnaru, Ismail M. Osmanov, Daniel Munblit, Sechenov StopCOVID Research Team, Nikol Alekseeva, Elena Artigas, Asmik Avagyan, Lusine Baziyants, Anna Belkina, Anna Berbenyuk, Tatiana Bezbabicheva, Vadim Bezrukov, Semyon Bordyugov, Aleksandra Borisenko, Maria Bratukhina, Ekaterina Bugaiskaya, Julia Chayka, Yulia Cherdantseva, Natalia Degtyareva, Olesya Druzhkova, Alexander Dubinin, Khalisa Elifkhanova, Dmitry Eliseev, Anastasia Ezhova, Aleksandra Frolova, Julia Ganieva, Anastasia Gorina, Cyrill Gorlenko, Elizaveta Gribaleva, Eliza Gudratova, Shabnam Ibragimova, Khadizhat Kabieva, Yulia Kalan, Margarita Kalinina, Nadezhda Khitrina, Bogdan Kirillov, Herman Kiseljow, Maria Kislova, Natalya Kogut, Irina Konova, Mariia Korgunova, Anastasia Kotelnikova, Karina Kovygina, Alexandra Krupina, Anastasia Kuznetsova, Anna Kuznetsova, Baina Lavginova, Elza Lidjieva, Ekaterina Listovskaya, Maria Lobova, Maria Loshkareva, Ekaterina Lyubimova, Daria Mamchich, Nadezhda Markina, Anastasia Maystrenko, Aigun Mursalova, Evgeniy Nagornov, Anna Nartova, Daria Nikolaeva, Georgiy Novoselov, Marina Ogandzhanova, Anna Pavlenko, Olga Perekosova, Erika Porubayeva, Kristina Presnyakova, Anna Pushkareva, Olga Romanova, Philipp Roshchin, Diana Salakhova, Ilona Sarukhanyan, Victoria Savina, Jamilya Shatrova, Nataliya Shishkina, Anastasia Shvedova, Denis Smirnov, Veronika Solovieva, Olga Spasskaya, Olga Sukhodolskaya, Shakir Suleimanov, Nailya Urmantaeva, Olga Usalka, Valeria Ustyan, Yana Valieva, Katerina Varaksina, Maria Varaksina, Ekaterina Varlamova, Maria Vodianova, Margarita Yegiyan, Margarita Zaikina, Anastasia Zorina, Elena Zuykova

**Affiliations:** 1 Laboratory of Health Economics, Institute of Applied Economic Studies, The Russian Presidential Academy of National Economy and Public Administration, Moscow, Russia; 2 Center for Advanced Financial Planning, Macroeconomic Analysis and Financial Statistics, Financial Research Institute of the Ministry of Finance of the Russian Federation, Moscow, Russia; 3 Department of Paediatrics and Paediatric Infectious Diseases, Institute of Child’s Health, Sechenov First Moscow State Medical University (Sechenov University), Moscow, Russia; 4 Tareev Clinic of Internal Diseases, Sechenov First Moscow State Medical University (Sechenov University), Moscow, Russia; 5 Clinic of Pulmonology, Sechenov First Moscow State Medical University (Sechenov University), Moscow, Russia; 6 Department of Internal Medicine №1, Institute of Clinical Medicine, Sechenov First Moscow State Medical University (Sechenov University), Moscow, Russia; 7 Research and Clinical Center for Neuropsychiatry, Moscow, Russia; 8 Pirogov Russian National Research Medical University, Moscow, Russia; 9 School of Applied Psychology, University College Cork, Cork City, Ireland; 10 Department of Clinical and Experimental Medicine, Section of Pediatrics, University of Pisa, Pisa, Italy; 11 Institute of Social Medicine and Health Systems Research, Faculty of Medicine, Otto von Guericke University Magdeburg, Magdeburg, Germany; 12 Pediatric Epidemiology, Department of Pediatrics, Medical Faculty, Leipzig University, Leipzig, Germany; 13 Universidad de La Sabana, Chía, Colombia; 14 Clínica Universidad de La Sabana, Chía, Colombia; 15 Department of Pediatric Pulmonology, Emma Children’s Hospital, Amsterdam University Medical Centers, Amsterdam, the Netherlands; 16 Department of Pediatrics, Tergooi MC, Hilversum, the Netherlands; 17 Sechenov First Moscow State Medical University (Sechenov University), Moscow, Russia; 18 Institute for Regenerative Medicine, Sechenov First Moscow State Medical University (Sechenov University), Moscow, Russia; 19 Russian Medical Academy of Continuous Professional Education of the Ministry of Healthcare of the Russian Federation, Moscow, Russia; 20 ZA Bashlyaeva Children’s Municipal Clinical Hospital, Moscow, Russia; 21 Research Institute for Healthcare Organization and Medical Management of Moscow Healthcare Department, Moscow, Russia; 22 Nuffield Department of Medicine, ISARIC Global Support Centre, University of Oxford, Oxford, UK; 23 MRC-University of Glasgow Centre for Virus Research, Glasgow, UK; 24 Department of Pediatrics, Section of Allergy/Immunology, Children’s Hospital Colorado, University of Colorado School of Medicine, Aurora, USA; 25 Department of Woman and Child Health and Public Health, Fondazione Policlinico Universitario A. Gemelli IRCCS, Rome, Italy; 26 Dipartimento di Scienze Biotecnologiche di Base, Cliniche Intensivologiche e Perioperatorie, Università Cattolica del Sacro Cuore, Rome, Italy; 27 Center for Global Health Research and Studies, Università Cattolica del Sacro Cuore, Roma, Italia; 28 Health Protection Research Unit in Emerging and Zoonotic Infections, Institute of Infection, Veterinary and Ecological Sciences, Faculty of Health and Life Sciences, University of Liverpool, Liverpool, UK; 29 Department of Respiratory Medicine, Alder Hey Children’s Hospital, Liverpool, UK; 30 Inflammation, Repair and Development Section, National Heart and Lung Institute, Faculty of Medicine, Imperial College London, London, UK; 31 Outcomes After Critical Illness and Surgery (OACIS) Research Group, Johns Hopkins University, Baltimore, MD USA; 32 Pulmonary and Critical Care Medicine, Department of Medicine, Johns Hopkins University School of Medicine, Baltimore, MD USA; 33 Physical Medicine and Rehabilitation, Johns Hopkins University School of Medicine, Baltimore, MD USA

**Keywords:** Adults, Children, COVID-19, COVID-19 sequelae, Long COVID, Post-acute sequelae of SARS-CoV-2 infection, PASC, Post-COVID-19 condition, Prevalence, Risk factor

## Abstract

**Background:**

Previous studies assessing the prevalence of COVID-19 sequelae in adults and children were performed in the absence of an agreed definition. We investigated prevalence of post-COVID-19 condition (PCC) (WHO definition), at 6- and 12-months follow-up, amongst previously hospitalised adults and children and assessed risk factors.

**Methods:**

Prospective cohort study of children and adults with confirmed COVID-19 in Moscow, hospitalised between April and August, 2020. Two follow-up telephone interviews, using the International Severe Acute Respiratory and Emerging Infection Consortium survey, were performed at 6 and 12 months after discharge.

**Results:**

One thousand thirteen of 2509 (40%) of adults and 360 of 849 (42%) of children discharged participated in both the 6- and 12-month follow-ups. PCC prevalence was 50% (95% CI 47–53) in adults and 20% (95% CI 16–24) in children at 6 months, with decline to 34% (95% CI 31–37) and 11% (95% CI 8–14), respectively, at 12 months. In adults, female sex was associated with PCC at 6- and 12-month follow-up (OR 2.04, 95% CI 1.57 to 2.65) and (OR 2.04, 1.54 to 2.69), respectively. Pre-existing hypertension (OR 1.42, 1.04 to 1.94) was associated with post-COVID-19 condition at 12 months. In children, neurological comorbidities were associated with PCC both at 6 months (OR 4.38, 1.36 to 15.67) and 12 months (OR 8.96, 2.55 to 34.82) while allergic respiratory diseases were associated at 12 months (OR 2.66, 1.04 to 6.47).

**Conclusions:**

Although prevalence of PCC declined one year after discharge, one in three adults and one in ten children experienced ongoing sequelae. In adults, females and persons with pre-existing hypertension, and in children, persons with neurological comorbidities or allergic respiratory diseases are at higher risk of PCC.

**Supplementary Information:**

The online version contains supplementary material available at 10.1186/s12916-022-02448-4.

## Background

Although most people fully recover from acute infection with severe acute respiratory syndrome coronavirus 2 (SARS CoV-2) and coronavirus disease 2019 (COVID-19) disease, some experience ongoing sequelae [1]. This wide range of symptoms occurring in the weeks to months after SARS-CoV-2 infection has been referred to as either long COVID, post-COVID-19 condition, or post-acute sequelae of SARS-CoV-2 infection (PASC), amongst other names [2]. High profile editorials [3, 4] drew attention to an increasing number of people experiencing these ongoing sequelae and called for comprehensive research, including risk factors and clinical features.

Most post-COVID research has focused on adults [5], given the predominance of adult COVID-19 in the first pandemic waves, which appeared to spare children, somewhat. Therefore, there is a more limited number of paediatric studies [6], although the need for research on COVID-19 consequences in children and young people has been previously acknowledged and has grown in importance with emergence of variants that are affecting children [7]. Head-to-head comparison of COVID-19 sequelae in children and adults is still lacking.

Many studies have investigated the prevalence and risk factors of long COVID [5], but heterogeneity in patient assessment and definitions [8] and lack of data regarding symptom duration are challenges to meta-analyses. Notably, in September 2020, the World Health Organization (WHO) Classification and Terminologies unit created International Classification of Diseases 10 (ICD-10) and ICD-11 codes for post-COVID-19 condition, and in October 2021, a clinical case definition of post-COVID-19 condition was announced, following a Delphi consensus process [9]. It was defined as a condition occurring “in individuals with a history of probable or confirmed SARS-CoV-2 infection, usually 3 months from the onset of COVID-19 with symptoms that last for at least 2 months and cannot be explained by an alternative diagnosis”. However, this definition was intended for adults, and WHO suggests that a separate definition might be applicable for children.

This prospective study aimed to investigate the prevalence and characteristics of post-COVID-19 condition in previously hospitalised children and adults using standardised follow-up data collection protocols developed by the International Severe Acute Respiratory and Emerging Infection Consortium (ISARIC) Global Adult and Paediatric COVID-19 follow-up working groups.

## Methods

The study is reported based on the Strengthening the Reporting of Observational Studies in Epidemiology (STROBE) checklist for cohort studies (https://www.strobe-statement.org/), which can be found in the supplementary material.

### Study design, setting, and participants

This study combines data from two longitudinal prospective cohorts of patients with COVID-19: (a) adults admitted to Sechenov University Hospital Network (four large tertiary adult hospitals) in Moscow, Russia, and (b) children admitted to Z.A. Bashlyaeva Children’s Municipal Clinical Hospital in Moscow, Russia (the primary paediatric COVID-19 hospital in Moscow throughout the time of pandemic). Only patients with positive polymerase chain reaction (PCR) confirmed SARS-CoV-2 infection were included in this study. Details regarding the demographic profile, hospitalisation requirements and origination of these cohorts are comprehensively described elsewhere [10, 11]. In brief, to form and define the cohorts, the acute phase data of adult and paediatric patients were extracted from electronic medical records (EMR) and the Local Health Information System (HIS) at the host institutions using ISARIC Core case report form (CRF) for acute phase data collection. The acute-phase datasets included demographics, comorbidities, symptoms on admission, computed tomography results, and disease severity, including use of supportive therapies.

Data collection and entry were performed by a team of trained medical students and physician residents, with extensive relevant research experience, via telephone interviews and the Research Electronic Data Capture (REDCap) database [10–12], with supervision by senior academic researchers.

Given the well-recognised emergence of COVID-19 infection sequelae, this follow-up study was planned to track prevalence and risk factors for the development of such sequelae occurring after hospital discharge. Data were obtained at two follow-up points, at 6 (± 2) and 12 (± 2) months after hospital discharge. These follow-up assessments, collected via telephone interviews, used the Tier 1 ISARIC Long-term Follow-up Study CRF for adult patients and version 1 of the ISARIC COVID-19 Health and Wellbeing Follow Up Survey for Children for paediatric patients, both developed by the ISARIC Global COVID-19 follow-up working group and independently forward and backward translated into Russian. These follow-up assessments evaluated patients’ physical and mental health status and assessed for any newly developed symptoms between hospital discharge and the follow-up assessment, including symptom onset and duration as previously described [11]. Given the well-recognised emergence of post-COVID infection sequelae, this follow-up study was planned to track prevalence and risk factors for the development of such sequelae occurring after hospital discharge.

The acute-hospitalisation dataset included demographics, symptoms, comorbidities (at the time of hospital admission for COVID-19), chest computed tomography (CT) results, supportive care required, and clinical outcomes at the time of discharge.

### Data management

REDCap electronic data capture tools (Vanderbilt University, Nashville, TN, USA) hosted at Sechenov University and Microsoft Excel (Microsoft Corp, Redmond, WA, USA) were used for data collection, storage, and management [13, 14].

### Definitions

Post-COVID-19 condition was defined as the presence of any symptom which started no later than three months after hospital discharge and lasted for at least 2 months as per the WHO case definition [9]. Symptom duration was calculated from the time of the hospital discharge in the absence of reliable objective medical record data regarding date of first symptoms appearance.

Patients requiring non-invasive ventilation, invasive ventilation, or intensive care unit (ICU) care during acute phase of COVID-19 were defined as severe.

Symptoms were categorised into nine manifestations: cardiovascular, dermatological, fatigue, gastrointestinal, musculoskeletal, neurocognitive, respiratory, sensory, and sleep (Table S1). Symptom categorisation was based on previously published literature and ISARIC working groups’ discussion [10, 11].

### Statistical analysis

Descriptive statistics were calculated for baseline characteristics. Continuous variables were summarised as median (interquartile range, IQR) and categorical variables as frequency (percentage). 95% confidence intervals (CIs) were obtained for the estimates of post-COVID-19 condition prevalence using bootstrap methodology (10,000 iterations).

Forest plots were used to present the prevalence of post-COVID-19 condition and different manifestations. Circular dendrograms were used to illustrate coexistence of post-COVID-19 condition manifestations. The phenotypes of post-COVID-19 condition were presented using radial plots. The cut-off for defining a phenotype presentation was set at 2% of respondents reporting multiple manifestations. For children, due to a low number of respondents reporting multiple manifestations, all individuals were presented on the plots.

We included all participants with post-COVID-19 condition in the final analysis, without missing data imputation. In order to control for recall bias at 12-month follow-up, we considered post-COVID-19 condition manifestations only amongst those manifestations which were reported at 6-months and satisfied the WHO definition of post-COVID-19 condition. Only patients completing both 6-month and 12-month follow-up were included in this study analysis similarly to previously published large cohort studies [15].

Multivariable logistic regression analysis was performed separately for adults and children to investigate associations of demographic characteristics and comorbidities at hospital admission with COVID-19 (limited to those variables reported in > 3% of study participants) and severity of COVID-19 with post-COVID-19 condition prevalence at the time of the follow-up interviews. Selection of the variables was the following: “COVID-19 severity” variable as exposure, “post-COVID-19 condition” as an outcome, comorbidities as covariates, gender, and age as effect modifiers. Twelve variables in adults and seven in children were tested as potential risk factors based on data availability and previous research [10, 11, 15–18]. We included all participants for whom the variables of interest were available in the final analysis, without imputing missing data. Odds ratios were calculated together with 95% CIs.

Two-sided *p*-values were reported for all statistical tests, a *p*-value below 0.05 was considered to be statistically significant. Statistical analysis was performed in R version 4.0.2 using libraries dplyr, foreign, forestplot, ograph, and ggraph [19].

## Results

**Fig. 1:**
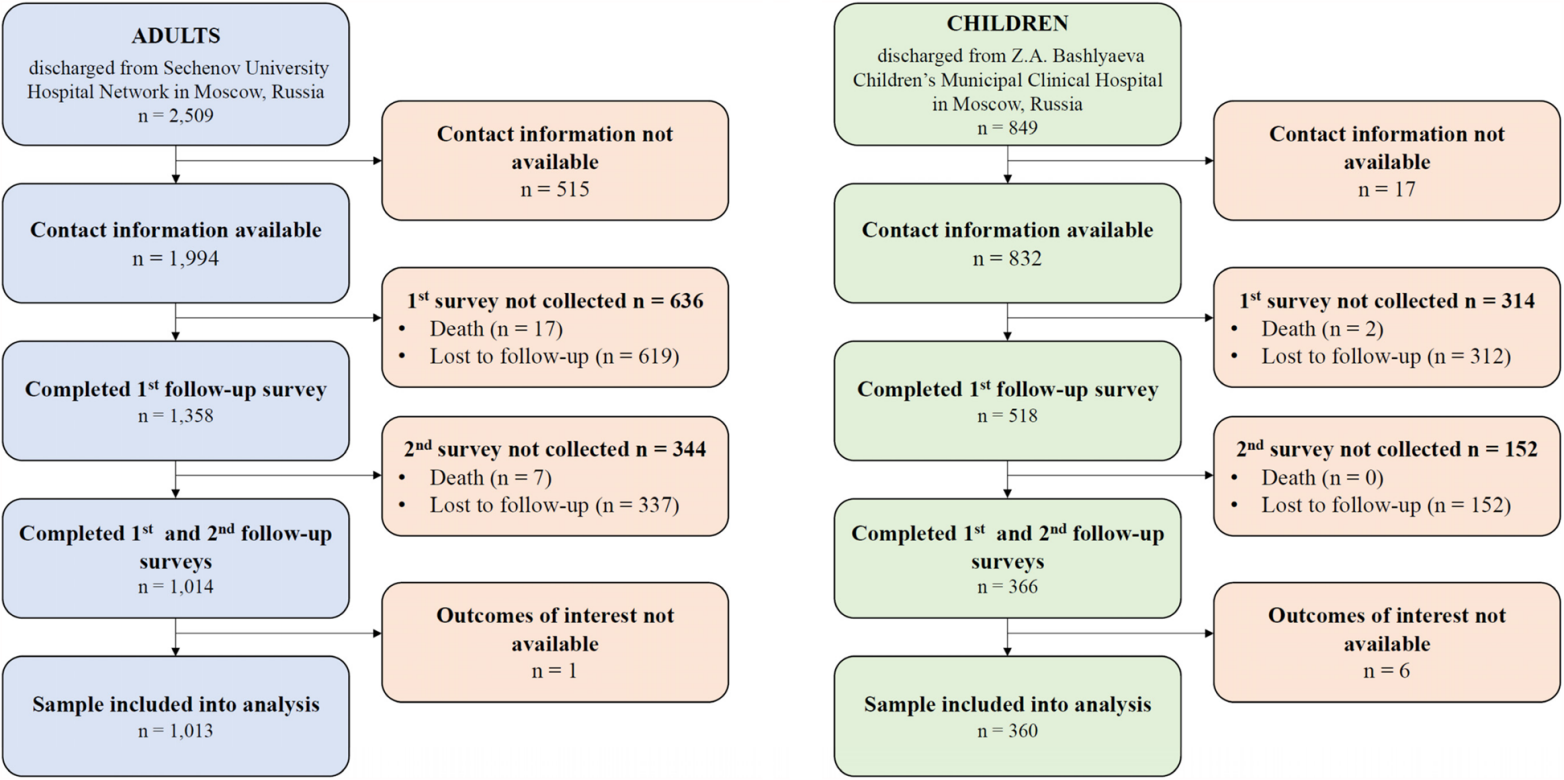
Flow diagram of patients admitted with PCR-confirmed COVID-19 to Sechenov University Hospital Network (adults) and Z.A. Bashlyaeva Children’s Municipal Clinical Hospital (children)

Out of 2509 eligible adults and 849 eligible children with laboratory confirmed COVID-19 discharged between April and August 2020, 1994 (79%) and 832 (98%) had contact information, and of these, 1013 (40% of discharged, 51% of those with contact information) adults and 360 children (42% of discharged, 43% of those with contact information) participated in both follow-up interviews and were included in the final analysis (Fig. 1). The 6-month follow-up interviews were conducted between November 2020 and March 2021 and the 12-month follow-up between April 2021 and August 2021.

Table 1 shows the demographic and clinical characteristics of the study participants. For adults the median time, after hospital discharge, to the 6- and 12-month assessments was 215 days (IQR 196–235) and 383 days (IQR 376–390). The median age of adult patients was 56.8 years (IQR 47.0–65.8), and 49% (500/1013) were male. The most common pre-existing comorbidity in adults at admission was hypertension (45%, 458/1013), followed by chronic cardiac disease and excessive weight and obesity (20% each, 198/1013) and type II diabetes (15%, 148/1013). Three percent of patients (27/1013) required non-invasive ventilation, invasive ventilation, or ICU care during hospitalisation.

**Table 1:** Demographic characteristics of adults admitted to the Sechenov University Hospital Network and children admitted to the Z.A. Bashlyaeva Children’s Municipal Clinical Hospital. Data are *n* (%) or median (IQR) excluding missing values. ICU, intensive care unit

Variable	Adults	Children
Number of participants	1013	360
Median age (IQR) at hospital admission, years	56.8 (47.0–65.8)	9.5 (2.4–14.8)
Median time from the hospital discharge to the 1st follow-up point (IQR), days	215 (196–235)	255 (223–270)
Median time from the hospital discharge to the 2nd follow-up point (IQR), days	383 (376–390)	367 (351–379)
Gender (female)	513/1013 (51%)	186/360 (52%)
Severe COVID-19 (requiring non-invasive ventilation or invasive ventilation or ICU)	27/1013 (3%)	12/360 (3%)
Heart diseases	502/1013 (49%)	12/360 (3%)
*Chronic cardiac disease*	198/1013 (20%)	N/A
*Hypertension*	458/1013 (45%)	N/A
*History of peripheral or cardiac revascularisation*	51/1013 (5%)	N/A
Respiratory diseases (not including asthma)	79/1013 (8%)	5/360 (1%)
Allergic respiratory diseases	N/A	29/360 (8%)
*Asthma (physician diagnosed)*	48/1013 (5%)	5/360 (1%)
*Allergic rhinitis/hay fever*	N/A	26/360 (7%)
Kidney disease	51/1013 (5%)	6/360 (2%)
Overweight and obesity (as defined by clinical staff)	198/1013 (20%)	10/360 (3%)
Neurological disorder	53/1013 (5%)	11/360 (3%)
Malignancy	42/1013 (4%)	0/360 (0%)
Haematological conditions	12/1013 (1%)	9/360 (3%)
Diabetes Mellitus	148/1013 (15%)	0/360 (0%)
Rheumatologic disorder	27/1013 (3%)	2/360 (1%)
Tuberculosis	1/1013 (0%)	3/360 (1%)
Malnutrition	1/1013 (0%)	10/360 (3%)
Intestinal (gut) problems	N/A	25/360 (7%)

For children, the median time to the 6-month follow-up was 255 days (IQR 223–270) and to the 12-month follow-up 367 days (IQR 351–379). The median paediatric patient age was 9.5 years (IQR 2.4–14.8), and 48% (174/360) were male. Three percent of children (12/360) required non-invasive ventilation, invasive ventilation, or treatment in the ICU during hospitalisation. The most common comorbidities in children were allergic rhinitis (7%, 26/360) and intestinal problems (7%, 25/360).

**Fig. 2:**
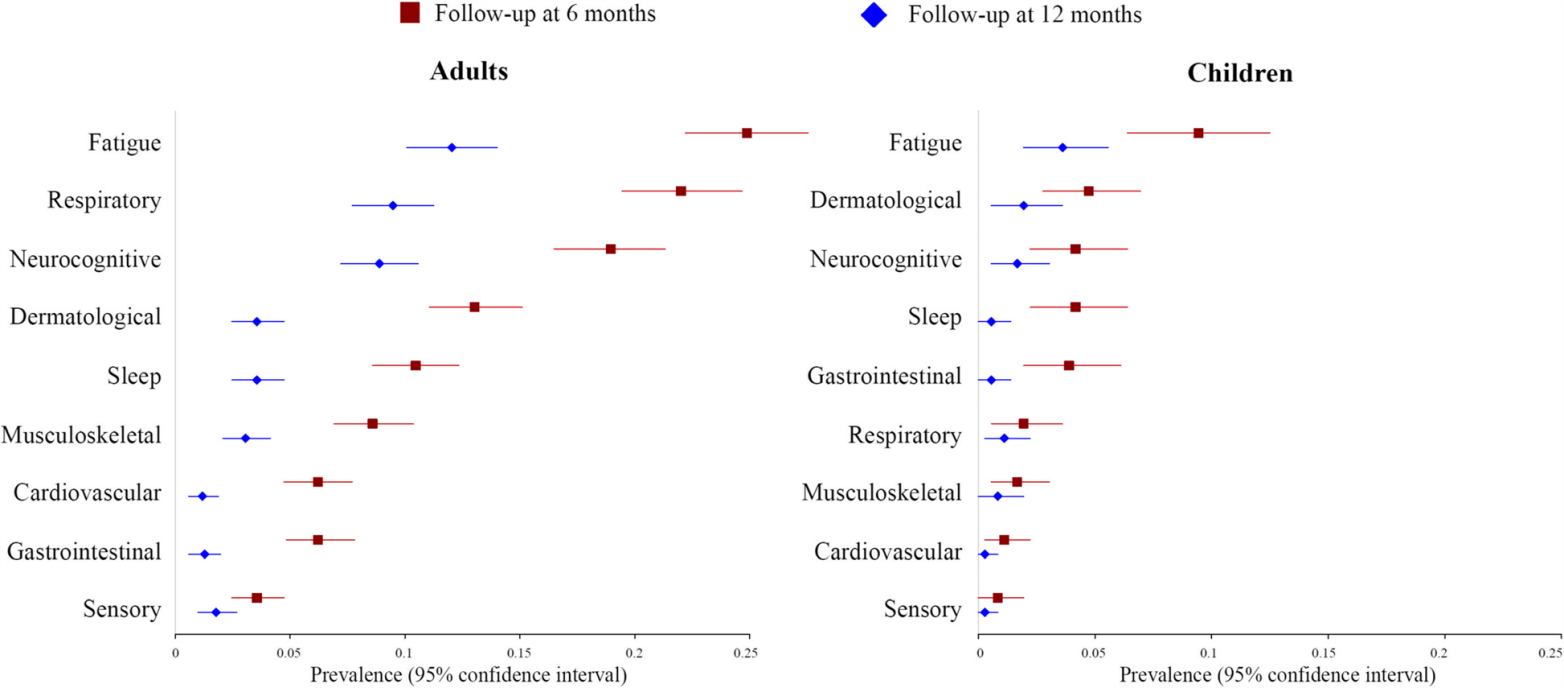
Forest plots demonstrating the prevalence of post-COVID-19 condition manifestations in adults and children 6 and 12 months after hospital discharge. Sixth-month prevalence is coloured in red, and 12-month prevalence is coloured in blue. Estimates of the prevalence 95% confidence intervals were calculated using the bootstrapping method

Figure 2 shows the temporal trend in post-COVID-19 condition manifestations prevalence. Prevalence was significantly higher in adults compared with children at both 6-month and 12-month follow-up (*p* < 0·001): relative risk of any manifestation 2.51 (2.02 to 3.11) at 6 months and 3.07 (2.26 to 4.16) at 12 months. The difference in prevalence of each specific manifestation between adults and children is shown in tables S2 and S3. The proportion of individuals with at least one post-COVID-19 condition manifestation decreased from 50% (95% CI 47–53) at 6 months to 34% (95% CI 31–37) at 12 months in adults and from 20% (95% CI 16–24) to 11% (95% CI 8–14), respectively, in children. A decline in prevalence was observed across all manifestations.

In adults, the most common post-COVID-19 condition features at 6-month follow-up included fatigue 25% (95% CI 22–28), respiratory 22% (95%CI 20–25), neuro-cognitive 19% (95% CI 17–21), and dermatological 13% (95% CI 11–15) manifestations. At 12 months after the hospital discharge, the prevalence decreased to 12% (95% CI 10–14), 10% (95% CI 8–11), 9% (95% CI 7–11), and 4% (95% CI 3–5) respectively.

In children, the most common post-COVID-19 condition features at 6-month follow-up were fatigue 9% (95% CI 6–13), dermatological 5% (95% CI 3–7), neuro-cognitive 4% (95% CI 2–6), and sleep-related 4% (95% CI 2–6) manifestations. At the 12-month follow-up, these decreased to 4% (95% CI 2–6), 2% (95% CI 1–4), 2% (95% CI 1–3), and 1% (95% CI 0–1) respectively.

**Fig. 3:**
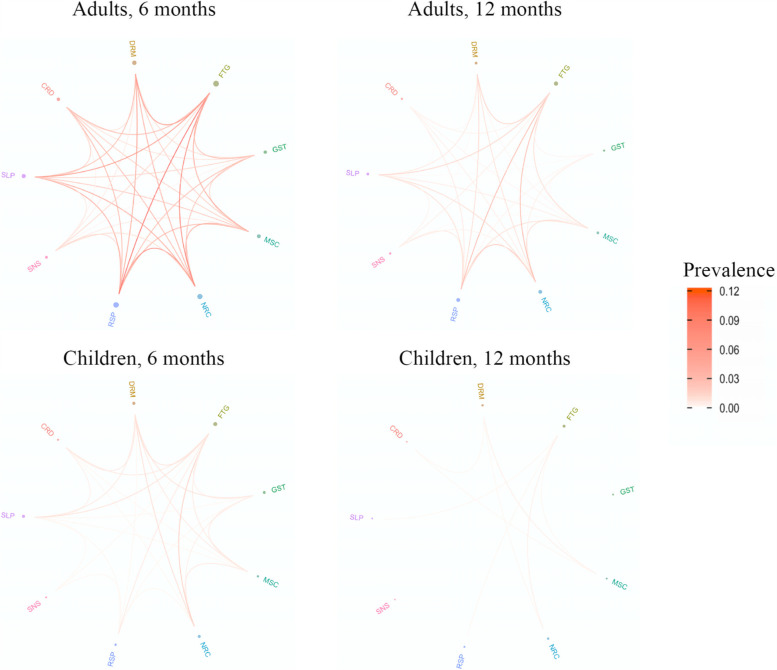
Interrelations between the post-COVID-19 condition manifestations in adults and children 6 and 12 months since hospital discharge. Bubble diameter is proportional to the proportion of individuals with the symptom category reported. Line thickness is proportional to the number of individuals with the coexisting manifestations. Cardiovascular, CRD; dermatological, DRM; fatigue, FTG; gastrointestinal, GST; musculoskeletal, MSC; neurocognitive, NRL; respiratory, RSP; sensory, SNS; sleep, SLP

**Fig. 4:**
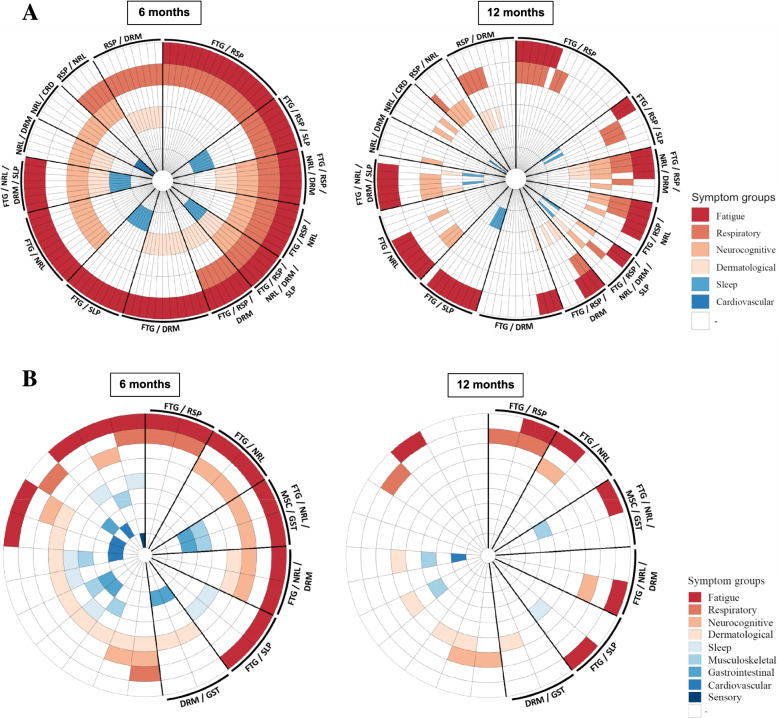
**A** Radial plots representing post-COVID-19 condition phenotypes in adults at 6 months after discharge and 12 months after discharge. Manifestations are shown for each patient; each segment represents a single patient. Thick black lines are used to distinct phenotypes. Cardiovascular, CRD; dermatological, DRM; fatigue, FTG; gastrointestinal, GST; musculoskeletal, MSC; neurocognitive, NRL; respiratory, RSP; sensory, SNS; sleep, SLP. **B** Radial plots representing post-COVID-19 condition phenotypes in children at 6 months after discharge and 12 months after discharge. Manifestations are shown for each patient; each segment represents a single patient. Thick black lines are used to distinct phenotypes. Cardiovascular, CRD; dermatological, DRM; fatigue, FTG; gastrointestinal, GST; musculoskeletal, MSC; neurocognitive, NRL; respiratory, RSP; sensory, SNS; sleep, SLP

We investigated the phenotypes of post-COVID-19 condition in adults and children, defined as a report of two or more different manifestations at 6-month assessment (Figs. 3 and 4). Amongst adults, 28% (287/1013) reported at least two manifestations. We differentiated three prevalent phenotypes at 6 months, namely (a) fatigue/respiratory without neurological manifestations (10%, 29/287); (b) fatigue/respiratory with neurological manifestations (7%, 19/287); and (c) fatigue/neurological without respiratory manifestations (6%, 17/287), regardless of other manifestations reported. By 12 months, 41% (12/29) of people with fatigue/respiratory without neurological manifestations fully recovered, while only 21% (4/19) of fatigue/respiratory with neurological manifestations and 24% (4/17) of fatigue/neurological without respiratory manifestations were symptom-free (Additional file 1: Figure S1).

In children, phenotypes were less feasible to assess due to a smaller case count of post-COVID-19 condition. Seven percent (25/360) of children had a combination of manifestations at 6-month follow-up. The only characteristic phenotype amongst individuals with coexisting manifestations was fatigue/neurological (24%, 6/25), with 50% (3/6) of these having fully resolved by 12 months.

**Fig. 5:**
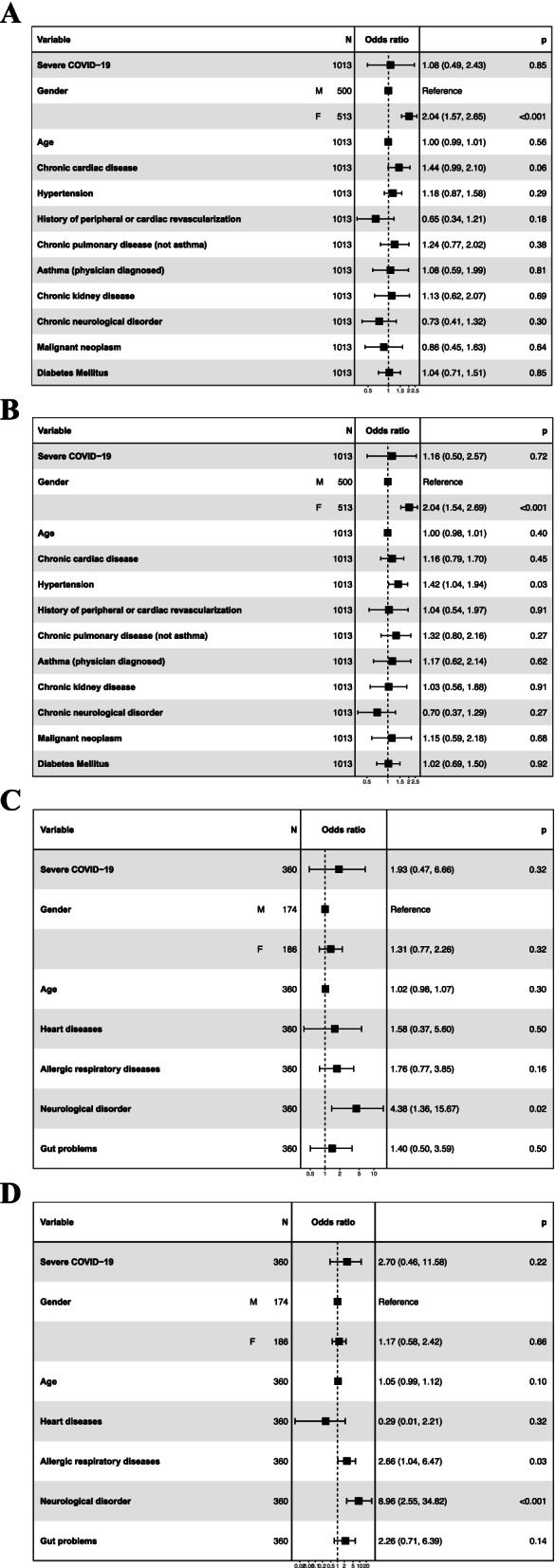
**A** Multivariable logistic regression model demonstrating risk factors associated with post-COVID-19 condition in adults at 6-month follow-up. Odds ratios and 95% CIs are presented. **B** Multivariable logistic regression model demonstrating risk factors associated with post-COVID-19 condition in adults at 12-month follow-up. Odds ratios and 95% CIs are presented. **C** Multivariable logistic regression model demonstrating risk factors associated with post-COVID-19 condition in children at 6-month follow-up. Odds ratios and 95% CIs are presented. **D** Multivariable logistic regression model demonstrating risk factors associated with post-COVID-19 condition in children at 12-month follow-up. Odds ratios and 95% CIs are presented

Risk factors association with post-COVID-19 condition 6- and 12-months after hospital discharge were assessed in multivariable regression analysis separately for adults and children (Fig. 5).

In adults, female sex was the only statistically significant risk factor of post-COVID-19 condition at both 6-month (odds ratio of 2.04 (95% CI 1.57 to 2.65) and 12-month (2.04, 1.54 to 2.69) follow-up. Pre-existing hypertension was also (1.42, 1.04 to 1.94) independently associated with post-COVID-19 condition at 12 months only.

In children, pre-existing neurological comorbidities were associated with post-COVID-19 condition at both 6-months (4.38, 1.36 to 15.67) and 12 months (8.96, 2.55 to 34.82). History of allergic respiratory diseases was a risk factor (2.66, 1.04 to 6.47) for post-COVID-19 condition at 12 months only.

## Discussion

This prospective cohort study with 1013 adults and 360 children, who were previously hospitalised with laboratory confirmed SARS-CoV-2 infection, assessed the 6- and 12-month prevalence of post-COVID-19 condition, according to the WHO case definition, along with phenotypes and risk factors. We found that half of adults and one of five children had post-COVID-19 condition at 6 months follow-up, with fatigue being the most common manifestation. Although prevalence of post-COVID-19 condition declined between 6- and 12-month assessment, one in three adults and one in ten children still had sequelae. Post-COVID-19 condition was experienced by both sexes, with a higher risk amongst adult women. Pre-existing hypertension (adults) and pre-existing neurological comorbidities and allergic respiratory diseases (children) were associated with post-COVID-19 condition.

The prevalence of post-COVID-19 condition was significantly higher in adults and the risk of post-COVID-19 condition was 2.5 and 3 times higher in adults relative to children 6 and 12 months post-hospital discharge, respectively. This was true also for individual symptom groups, except gastrointestinal at 6 months, and cardiovascular, dermatological, and gastrointestinal at 12 months. Although persistent symptoms of COVID-19 have been assessed in many studies [5], most published research was performed prior to the WHO post-COVID-19 case definition [9] in absence of agreed terminology and associated data heterogeneity. With differences in methodology, outcome definitions, and absence of symptom duration measurement across cohorts, it is difficult to evaluate the prevalence of post-COVID-19 condition. Another limitation in the existing literature is the inadequate knowledge of COVID-19 sequelae in children [6] and the lack of direct head-to-head comparison of its features and prevalence in adults and children, which do not allow for complete understanding if manifestations behave differently based on age.

Persistence of symptoms is a worrisome issue, with half of the adults in our study reported post-COVID-19 condition at 6 months, and 34% still experiencing one or more manifestations 12 months after discharge. This finding is consistent with data from China, which reported a high rate of single sequelae symptom prevalence and decrease from 68% at 6 months to 49% at 12 months [15]. Difference in prevalence may be related differences in post-COVID condition definitions, as the Chinese study was published before the WHO case definition announcement. We found a twofold decrease in the prevalence of post-COVID-19 condition from 20% between 6 and 12 months in children. To our knowledge, this is the first study reporting consequences of COVID-19 in children 1 year after acute episode, though we did detect persistent symptoms 6 months after hospital discharge in children in a previous study especially in older children and those with allergic disease [11]. Fatigue was the most common manifestation in both children and adults, regardless of the follow-up time point, though proportionally more adults than children reported fatigue. This finding is consistent with prior data [5, 6]. In adults and children, respiratory manifestations were reported in 20% and 2%, respectively. This difference may be related to greater severity of viral pneumonia in adults, as well as greater baseline respiratory comorbidity [20]. More frequent incidental infection rates may also confer some degree of non-specific immunological protection but the association with pre-existing respiratory allergy suggests that allergic hypersensitivity and/or auto-immune responses are involved. More research into pathophysiology and immune mechanisms is required to establish the cause of described association.

One third of individuals with post-COVID-19 condition can be classified by a phenotypes, of combined manifestations. One in five can be characterised by a combination of fatigue and respiratory with or without neurological manifestations. These results are similar to those reported by Taquet and colleagues [21]. We found that people without neurological manifestations become asymptomatic by 12 months more frequently than those reporting neurological manifestations at 6 months. However, due to a limited number of individuals available for phenotyping, it is premature to make any definitive conclusions.

The risk of post-COVID-19 condition was twice as high in female as in male adult patient at both time points, in line with previous studies assessing persistent symptoms [15, 17, 22]. Pre-existing hypertension was associated with post-COVID-19 condition at 12 months in adults. The association between pre-existing hypertension and higher risk of post-COVID-19 condition 12 months after hospital discharge in adults has not been previously reported [23], which may be explained by the difference in outcome definition. Pre-existing neurological comorbidities and allergic respiratory diseases were associated with post-COVID-19 in children. While allergic diseases are felt to be protective of developing COVID-19, this may become a risk factor for the sequelae development and merits further consideration. It was previously hypothesised that allergic conditions may increase the risk of long-term consequences following COVID-19 and that eosinophils, mast cells, or Th-2 responses may be potentially involved in the immunopathology of post-COVID-19 condition [24], but large prospective studies with biological material collection are required to confirm this.

This study has both strengths and limitations. Strengths include the following: (1) using of standardised ISARIC Long-term Follow-up Study CRFs for adults and children; (2) using the WHO post-COVID-19 condition definition; (3) enrolling both adults and children and comparing the two cohorts; and (4) a relatively large sample size of people attending both the 6- and 12-month follow-up visits, one of the longest follow-up assessments of hospitalised patients to-date. Limitations include the following: (1) questions about spectrum composition, as we enrolled only patients from Moscow (which may limit generalisability), a low proportion of whom had severe COVID-19—issues shared with most major COVID-19 cohort studies. This limitation is balanced by their otherwise being a paucity of data from eastern Europe regarding any COVID-19 outcomes, which becomes a novelty; (2) acute data were collected from the electronic medical records with no access to additional information that could be potentially retrieved from the medical notes—we mitigated potential inaccuracies of demographic information reported by the patients/parents/carers at the time of the hospital admission with subsequent verification during follow-up telephone interviews. This is an accepted and common limitation of cohorts assembled using this methodology; (3) a low proportion of patients with severe COVID-19 patients amongst both adults and children in our cohort limits the generalisability of the study findings to hospitalised patients with more mild to moderate COVID-19; (4) parents/caregivers were interviewed in this study and not children themselves, which is an accepted limitation of paediatric research conducted in children of a particular age; (5) a risk of potential selection bias, for instance with those with symptoms more likely to agree to survey and thus overestimating the prevalence of post-COVID-19 condition [25], as only 68% of adults and 62% of children for whom we had contact information agreed to participate in our study, and 51% and 42% respectively completing both visits—although retention of over 40% is generally considered good and rates here are comparable or higher than in the recent similar cohort studies [15, 26]. Any attrition from a cohort may result in a substantial overestimation of the prevalence of post-COVID-19 condition as those who do remain in the cohort may represent a biased sample [25]. However, we did not find significant differences between respondents and non-respondents (Table S4); (6) telephone interviews were used in this study, and we acknowledge that face-to-face interviews and/or objective measurements would deliver more robust results. However, financial and pandemic restrictions did not allow for this; (7) the study used hospitalised patients’ data. Interpretation of the data gathered from such sample may be prone to collider bias, as the sample is non-random as is conditions on hospital admission.

We used the ISARIC/WHO Clinical Characterisation Protocol, a prospective pandemic preparedness protocol which is agnostic to disease and has a pragmatic design to allow recruitment during pandemic conditions. As we already underlined in previous publications, the reality of conducting research in outbreak conditions is such that appropriate co-enrolment of a control group is practically challenging, primarily because COVID-19 has overshadowed other infections which could be used as comparators, and because of the lack of agreement on a commonly accepted control group [10].

## Conclusions

This study has shown that half of adults and one of five children have post-COVID-19 condition, as per WHO case definition, 6 months after hospital discharge, with fatigue being the most common manifestation. Respiratory manifestations also were a major problem in adults. Although the prevalence of post-COVID-19 condition declined, one in three adults and one in ten children still had manifestations at 12 months follow-up. Post-COVID-19 condition was more common adult women and amongst adults with pre-existing hypertension. In children, pre-existing neurological comorbidities and allergic respiratory diseases were associated with post-COVID-19 condition. Future studies should define COVID-19 as per the new WHO case definition to allow for a better comparability. Further investigation of risk factors and underlying physiological and immunological mechanisms merit further consideration.

## Supplementary Information

**Additional file 1: Table S1.** Categorisation of symptoms. **Table S2.** Prevalence of post-COVID-19 condition manifestations in adults and children at first (6 months) and second (12 months) follow-up. **Table S3.** *P* values for prevalence of post-COVID-19 condition manifestations comparison between adults and children at first (6 months) and second (12 months) follow-up. **Table S4.** Characteristics (comorbidities and COVID-19 severity) among respondents and non-respondents (adults and children) at first (6 months) and second (12 months) follow-up. **Figure S1.** Post-COVID-19 condition phenotypes in adults at first (6 month) follow-up and subsequent complete resolving by 12 months.

## Data Availability

The data that support the findings of this study are available from the corresponding author, DM, upon reasonable request.

## Abbreviations

CI: Confidence interval
COVID-19: Coronavirus disease 2019
CRF: Case report form
EMR: Electronic medical records
HIS: Local Health Information System
ISARIC: International Severe Acute Respiratory and Emerging Infection Consortium
ICD: International Classification of Diseases
ICU: Intensive care unit
IQR: Interquartile range
OR: Odds ratio
PASC: Post-acute sequelae of SARS-CoV-2 infection
PCC: Post-COVID-19 condition
PCR: Polymerase chain reaction
REDCap: Research Electronic Data Capture
SARS CoV-2: Severe acute respiratory syndrome-related coronavirus 2
STROBE: Strengthening the Reporting of Observational Studies in Epidemiology
WHO: World Health Organization
