# Supplementary material for: Prevalence and risk factors of post-COVID-19 condition in adults and children at 6 and 12 months after hospital discharge: a prospective, cohort study in Moscow (StopCOVID)
Source: BMC Med. 2022 Jul 6;20:244. doi: 10.1186/s12916-022-02448-4 (PMC9257572; doi:10.1186/s12916-022-02448-4)

**Prevalence and risk factors of post-COVID-19 condition in adults and children at 6 and 12 months after hospital discharge: a prospective, cohort study in Moscow (Stop COVID)**

**Supplementary Material**

# Ekaterina Pazukhina MSc^1,2^*, Margarita Andreeva BSc^3^*, Ekaterina Spiridonova BSc^3^*, Polina Bobkova BSc^3^*, Anastasia Shikhaleva BSc^3^*, Yasmin El-Taravi BSc^3^*, Mikhail Rumyantsev BSc^3^, Anastasiia Bairashevskaia BSc^3^, Aysylu Gamirova BSc^3^*, Dina Baimukhambetova BSc^3^, Maria Pikuza BSc^3^, Elina Abdeeva BSc^3^, Yulia Filippova BSc^3^, Polina Petrova BSc^3^, Salima Deunezhewa BSc^3^, Nikita Nekliudov MD^3^, Polina Bugaeva MD^3^, Nikolay Bulanov MD PhD^4^, Sergey Avdeev MD PhD^5^, Valentina Kapustina MD PhD^6^, Alla Guekht MD PhD^7,8^, Audrey DunnGalvin PhD^3,9^, Pasquale Comberiati MD^10,11^, Diego G Peroni MD PhD^11^, Christian Apfelbacher PhD PhD^12^, Jon Genuneit MD PhD^13^, Luis Felipe Reyes MD PhD^14,15^, Caroline LH Brackel MD^16,17^, Victor Fomin MD PhD^18^, Andrey A Svistunov MD PhD^18^, Peter Timashev PhD^19^, Lyudmila Mazankova MD PhD^20^, Alexandra Miroshina MD PhD^21^, Elmira Samitova MD PhD^20,21^, Svetlana Borzakova MD PhD^8,22^, Elena Bondarenko MD^3^, Anatoliy A Korsunskiy MD PhD^3^, Gail Carson MD PhD^23^, Louise Sigfrid MD PhD^23^, Janet T Scott MD PhD^24^, Matthew Greenhawt MD MBA^25^, Danilo Buonsenso MD^26,27,28^, Malcolm G Semple MD PhD^29,30^, John O Warner MD FMedSci^31^, Piero Olliaro MD PhD^23^, Dale M Needham MD PhD^32,33,34^, Petr Glybochko MD PhD^18^, Denis Butnaru MD PhD^18^, Ismail M Osmanov MD PhD^8,21^*, Daniel Munblit MD PhD^3,7,31^* and Sechenov StopCOVID Research Team

1. Laboratory of Health Economics, Institute of Applied Economic Studies, The Russian Presidential Academy of National Economy and Public Administration, Moscow, Russia
2. Center for Advanced Financial Planning, Macroeconomic Analysis and Financial Statistics, Financial Research Institute of the Ministry of Finance of the Russian Federation, Moscow, Russia
3. Department of Paediatrics and Paediatric Infectious Diseases, Institute of Child’s Health, Sechenov First Moscow State Medical University (Sechenov University), Moscow, Russia
4. Tareev Clinic of Internal Diseases, Sechenov First Moscow State Medical University (Sechenov University), Moscow, Russia
5. Clinic of Pulmonology, Sechenov First Moscow State Medical University (Sechenov University), Moscow, Russia
6. Department of Internal Medicine №1, Institute of Clinical Medicine, Sechenov First Moscow State Medical University (Sechenov University), Moscow, Russia
7. Research and Clinical Center for Neuropsychiatry, Moscow, Russia
8. Pirogov Russian National Research Medical University, Moscow, Russia
9. School of Applied Psychology, University College Cork, Cork City, Ireland
10. Department of Clinical Immunology and Allergology, Sechenov First Moscow State Medical University (Sechenov University), Moscow, Russia
11. Department of Clinical and Experimental Medicine, Section of Pediatrics, University of Pisa, Pisa, Italy
12. Institute of Social Medicine and Health Systems Research, Faculty of Medicine, Otto von Guericke University Magdeburg, Magdeburg, Germany
13. Pediatric Epidemiology, Department of Pediatrics, Medical Faculty, Leipzig University, Leipzig, Germany
14. Universidad de La Sabana, Chía, Colombia
15. Clínica Universidad de La Sabana, Chía, Colombia
16. Department of Pediatric Pulmonology, Emma Children's Hospital, Amsterdam University Medical Centers, Amsterdam, the Netherlands
17. Department of Pediatrics, Tergooi MC, Hilversum, the Netherlands
18. Sechenov First Moscow State Medical University (Sechenov University), Moscow, Russia
19. Institute for Regenerative Medicine, Sechenov First Moscow State Medical University (Sechenov University, Moscow, Russia
20. Russian Medical Academy of Continuous Professional Education of the Ministry of Healthcare of the Russian Federation, Moscow, Russia
21. ZA Bashlyaeva Children's Municipal Clinical Hospital, Moscow, Russia
22. Research Institute for Healthcare Organization and Medical Management of Moscow Healthcare Department, Moscow, Russia
23. Nuffield Department of Medicine, ISARIC Global Support Centre, University of Oxford, Oxford, UK
24. MRC-University of Glasgow Centre for Virus Research, Glasgow, UK
25. Department of Pediatrics, Section of Allergy/Immunology, Children's Hospital Colorado, University of Colorado School of Medicine, United States
26. Department of Woman and Child Health and Public Health, Fondazione Policlinico Universitario A. Gemelli IRCCS, Rome, Italy
27. Dipartimento di Scienze Biotecnologiche di Base, Cliniche Intensivologiche e Perioperatorie, Università Cattolica del Sacro Cuore, Rome, Italy
28. Center for Global Health Research and Studies, Università Cattolica del Sacro Cuore, Roma, Italia
29. Health Protection Research Unit in Emerging and Zoonotic Infections, Institute of Infection, Veterinary and Ecological Sciences, Faculty of Health and Life Sciences, University of Liverpool, Liverpool, UK
30. Department of Respiratory Medicine, Alder Hey Children's Hospital, Liverpool, UK
31. Inflammation, Repair and Development Section, National Heart and Lung Institute, Faculty of Medicine, Imperial College London, London, United Kingdom
32. Outcomes After Critical Illness and Surgery (OACIS) Research Group, Johns Hopkins University, Baltimore MD, United States
33. Pulmonary and Critical Care Medicine, Department of Medicine, Johns Hopkins University School of Medicine, Baltimore MD, United States
34. Physical Medicine and Rehabilitation, Johns Hopkins University School of Medicine, Baltimore MD, United States

*Authors contributed equally to the paper.

Table of Contents

[Supplementary Materials 4](#_Toc94198249)

[Sechenov Stop COVID Research Team (Group authors): 4](#_Toc94198250)

[Table S1 Categorisation of symptoms 5](#_Toc94198251)

[Table S2 Prevalence of post-COVID-19 condition manifestations in adults and children at first (6 months) and second (12 months) follow-up. 6](#_Toc94198252)

[Table S3 P values for prevalence of post-COVID-19 condition manifestations comparison between adults and children at first (6 months) and second (12 months) follow-up. 7](#_Toc94198253)

[Figure S1 Post-COVID-19 condition phenotypes in adults at first (6 month) follow-up and subsequent complete resolving by 12 months. 8](#_Toc94198254)

# **Supplementary Materials**

## **Sechenov Stop COVID Research Team (Group authors):**

Nikol Alekseeva¹, Elena Artigas¹, Asmik Avagyan¹, Lusine Baziyants¹, Anna Belkina¹, Anna Berbenyuk¹, Tatiana Bezbabicheva¹, Vadim Bezrukov¹, Semyon Bordyugov¹, Aleksandra Borisenko¹, Maria Bratukhina¹, Ekaterina Bugaiskaya¹, Julia Chayka¹, Yulia Cherdantseva¹, Natalia Degtyareva¹, Olesya Druzhkova¹, Alexander Dubinin¹, Khalisa Elifkhanova¹, Dmitry Eliseev¹, Anastasia Ezhova¹, Aleksandra Frolova¹, Julia Ganieva¹, Anastasia Gorina¹, Cyrill Gorlenko¹, Elizaveta Gribaleva¹, Eliza Gudratova¹, Shabnam Ibragimova¹, Khadizhat Kabieva¹, Yulia Kalan¹, Margarita Kalinina¹, Nadezhda Khitrina², Bogdan Kirillov¹, Herman Kiseljow¹, Maria Kislova¹, Natalia Kogut¹, Irina Konova^3^, Mariia Korgunova¹, Anastasia Kotelnikova¹, Karina Kovygina¹, Alexandra Krupina¹, Anastasia Kuznetsova¹, Anna Kuznetsova¹, Baina Lavginova¹, Elza Lidjieva¹, Ekaterina Listovskaya¹, Maria Lobova¹, Maria Loshkareva¹, Ekaterina Lyubimova¹, Daria Mamchich¹, Nadezhda Markina¹, Anastasia Maystrenko¹, Aigun Mursalova¹, Evgeniy Nagornov¹, Anna Nartova¹, Daria Nikolaeva¹, Georgiy Novoselov¹, Marina Ogandzhanova¹, Anna Pavlenko¹, Olga Perekosova¹, Erika Porubayeva¹, Kristina Presnyakova¹, Anna Pushkareva¹, Olga Romanova¹, Philipp Roschin¹, Diana Salakhova¹, Ilona Sarukhanyan¹, Victoria Savina¹, Jamilya Shatrova¹, Nataliya Shishkina¹, Anastasia Shvedova¹, Denis Smirnov¹, Veronika Solovieva¹, Olga Spasskaya¹, Olga Sukhodolskaya¹, Shakir Suleimanov¹, Nailya Urmantaeva¹, Olga Usalka¹, Valeria Ustyan¹, Yana Valieva¹, Katerina Varaksina¹, Maria Varaksina¹, Ekaterina Varlamova¹, Maria Vodianova¹, Margarita Yegiyan¹, Margarita Zaikina¹, Anastasia Zorina¹, Elena Zuykova¹

¹ Sechenov First Moscow State Medical University (Sechenov University), Moscow, Russia

² 1C First Bit, Moscow, Russia

^3^ ZA Bashlyaeva Children's Municipal Clinical Hospital, Moscow, Russia

***The names of the authors are in alphabetic order***

## **Table S1** Categorisation of symptoms

| **№** | **Abbreviation** | **Category** | **Symptoms** |
| --- | --- | --- | --- |
| 1 | MSC | Musculoskeletal | Joint pain or swelling |
|  |  |  | Persistent muscle pain |
| 2 | CRD | Cardiovascular | Palpitations (heart racing) |
| 3 | RSP | Respiratory | Shortness of breath/breathlessness |
|  |  |  | Pain on breathing |
|  |  |  | Persistent cough |
|  |  |  | Chest pain |
| 4 | NRL | Neurological and cognitive dysfunction | Cannot fully move or control movement |
|  |  |  | Problems with balance |
|  |  |  | Tremor/shakiness |
|  |  |  | Seizures |
|  |  |  | Tingling feeling/ “pins and needles“ |
|  |  |  | Confusion/lack of concentration |
|  |  |  | Problems speaking or communicating |
|  |  |  | Fainting/ blackouts |
|  |  |  | Dizziness/ light headedness |
|  |  |  | Problems seeing/blurred vision |
|  |  |  | Forgetfulness (adults only) |
| 5 | DRM | Dermatological | Hair loss |
|  |  |  | Skin rash |
|  |  |  | Hyperhidrosis |
| 6 | GST | Gastrointestinal | Constipation |
|  |  |  | Diarrhoea |
|  |  |  | Stomach/ abdominal pain |
|  |  |  | Vomiting |
| 7 | SNS | Sensory | Loss of smell |
|  |  |  | Loss of taste |
| 8 | SLP | Sleep | Problems sleeping |
|  |  |  | Hypersomnia (children only) |
| 9 | FTG | Fatigue | Fatigue |

## **Table S2** Prevalence of post-COVID-19 condition manifestations in adults and children at first (6 months) and second (12 months) follow-up.

| **post-COVID-19**  **condition manifestations** | **6-month follow-up** | | | | | |
| --- | --- | --- | --- | --- | --- | --- |
|  | **Adults** | | | **Children** | | |
|  | **Mean prevalence** | **Lower CI** | **Upper CI** | **Mean prevalence** | **Lower CI** | **Upper CI** |
| FTG | 252/1013 (24.88%) | 22.21% | 27.54% | 34/360 (9.44%) | 6.39% | 12.5% |
| RSP | 223/1013 (22.01%) | 19.45% | 24.68% | 7/360 (1.94%) | 0.56% | 3.61% |
| NRL | 192/1013 (18.95%) | 16.49% | 21.32% | 15/360 (4.17%) | 2.22% | 6.39% |
| DRM | 132/1013 (13.03%) | 11.06% | 15.1% | 17/360 (4.72%) | 2.78% | 6.94% |
| SLP | 106/1013 (10.46%) | 8.59% | 12.34% | 15/360 (4.17%) | 2.22% | 6.39% |
| MSC | 87/1013 (8.59%) | 6.91% | 10.37% | 6/360 (1.67%) | 0.56% | 3.06% |
| CRD | 63/1013 (6.22%) | 4.74% | 7.7% | 4/360 (1.11%) | 0.28% | 2.22% |
| GST | 63/1013 (6.22%) | 4.84% | 7.8% | 14/360 (3.89%) | 1.94% | 6.11% |
| SNS | 36/1013 (3.55%) | 2.47% | 4.74% | 3/360 (0.83%) | 0% | 1.94% |
| Any manifestation | 508/1013 (50.15%) | 47.09% | 53.31% | 72/360 (20%) | 15.83% | 24.17% |
|  | **12-month follow-up** | | | | | |
|  | **Adults** | | | **Children** | | |
| FTG | 122/1013 (12.04%) | 10.07% | 14.02% | 13/360 (3.61%) | 1.94% | 5.56% |
| RSP | 96/1013 (9.48%) | 7.7% | 11.25% | 4/360 (1.11%) | 0.28% | 2.22% |
| NRL | 90/1013 (8.88%) | 7.21% | 10.56% | 6/360 (1.67%) | 0.56% | 3.06% |
| DRM | 36/1013 (3.55%) | 2.47% | 4.74% | 7/360 (1.94%) | 0.56% | 3.61% |
| SLP | 36/1013 (3.55%) | 2.47% | 4.74% | 2/360 (0.56%) | 0% | 1.39% |
| MSC | 31/1013 (3.06%) | 2.07% | 4.15% | 3/360 (0.83%) | 0% | 1.94% |
| SNS | 18/1013 (1.78%) | 0.99% | 2.67% | 1/360 (0.28%) | 0% | 0.83% |
| GST | 13/1013 (1.28%) | 0.59% | 1.97% | 2/360 (0.56%) | 0% | 1.39% |
| CRD | 12/1013 (1.18%) | 0.59% | 1.88% | 1/360 (0.28%) | 0% | 0.83% |
| Any manifestation | 345/1013 (34.06%) | 31.19% | 36.92% | 40/360 (11.11%) | 8.06% | 14.44% |

## **Table S3** P values for prevalence of post-COVID-19 condition manifestations comparison between adults and children at first (6 months) and second (12 months) follow-up.

| **Symptom group** | **P-value**  **6-month follow-up** | **P-value**  **12-month follow-up** |
| --- | --- | --- |
| MSC | 0.0000*** | 0.0325* |
| CRD | 0.0000*** | **0.2034** |
| RSP | 0.0000*** | 0.0000*** |
| NRL | 0.0000*** | 0.0000*** |
| DRM | 0.0000*** | **0.1593** |
| GST | 0.1292 | **0.3786** |
| SNS | 0.0130* | 0.0674 |
| SLP | 0.0004*** | 0.0052** |
| FTG | 0.0000*** | 0.0000*** |
| Any manifestation | 0.0000*** | 0.0000*** |

Table - Difference between prevalence of symptoms among children and adults by the points of survey

P-values were computed using Chi square test except where highlighted in bold (due to low expected frequency exact Fisher's test is used to compute p-values).

## **Table S4** Characteristics (comorbidities and COVID-19 severity) among respondents and non-respondents (adults and children) at first (6 months) and second (12 months) follow-up.

| Variable | Respondents | | Non-respondents | | P-value | |
| --- | --- | --- | --- | --- | --- | --- |
|  | Adults | Children | Adults | Children | Adults | Children |
| Median age (IQR) at hospital admission, years | 56.8 (47.0 - 65.8) | 9.5  (2.4 - 14.8) | 57.6  (47.3 - 66.9) | 11.5  (3.7 - 16.2) |  |  |
| Gender (female) | 50.6% | 51.7% | 49.3% | 52.8% |  |  |
| Severe COVID-19 (requiring non-invasive ventilation or invasive ventilation or ICU) | 2.7% | 3.3% | 2.9% | 1.8% | 0.84 | 0.77 |
| Heart diseases |  | 3% |  | 4% |  | 0.80 |
| *Chronic cardiac disease* | *20%* |  | *22%* |  | 0.70 |  |
| *Hypertension* | *45%* |  | *49%* |  | 0.17 |  |
| *History of peripheral or cardiac revascularization* | *5%* |  | *3%* |  | 0.99 |  |
| Respiratory diseases (not including asthma) | 8% |  | 8% |  |  | 0.26 |
| Asthma (physician diagnosed) | 5% | 1% | 4% | 2% | 0.66 | 0.99 |
| Allergic rhinitis/hay fever |  | 7% |  | 10% |  | 0.31 |
| Allergic respiratory diseases |  | 8% |  | 10% |  | 0.88 |
| Kidney disease | 5% | 2% | 6% | 2% | 0.41 | 0.60 |
| Overweight and obesity (as defined by clinical staff) | 20% | 3% | 19% | 2% | 0.48 | 0.65 |
| Neurological disorder | 5% | 3% | 7% | 8% | 0.28 | 0.31 |
| Malignancy | 4% |  | 4% |  | 0.18 |  |
| Malnutrition |  | 3% |  | 4% |  | 0.73 |
| Intestinal(gut) problems |  | 7% |  | 8% |  | 0.86 |
| Haematological conditions | 1% | 3% | 1% | 2% | 0.08 | 0.99 |
| Diabetes Mellitus | 15% |  | 15% |  | 0.60 |  |
| Rheumatologic disorder | 3% | 1% | 3% | 1% | 0.15 | 0.58 |
| Tuberculosis | 0% | 1% | 0% | 4% | 0.99 | 0.29 |

## **Figure S1** Post-COVID-19 condition phenotypes in adults at first (6 month) follow-up and subsequent complete resolving by 12 months.

##
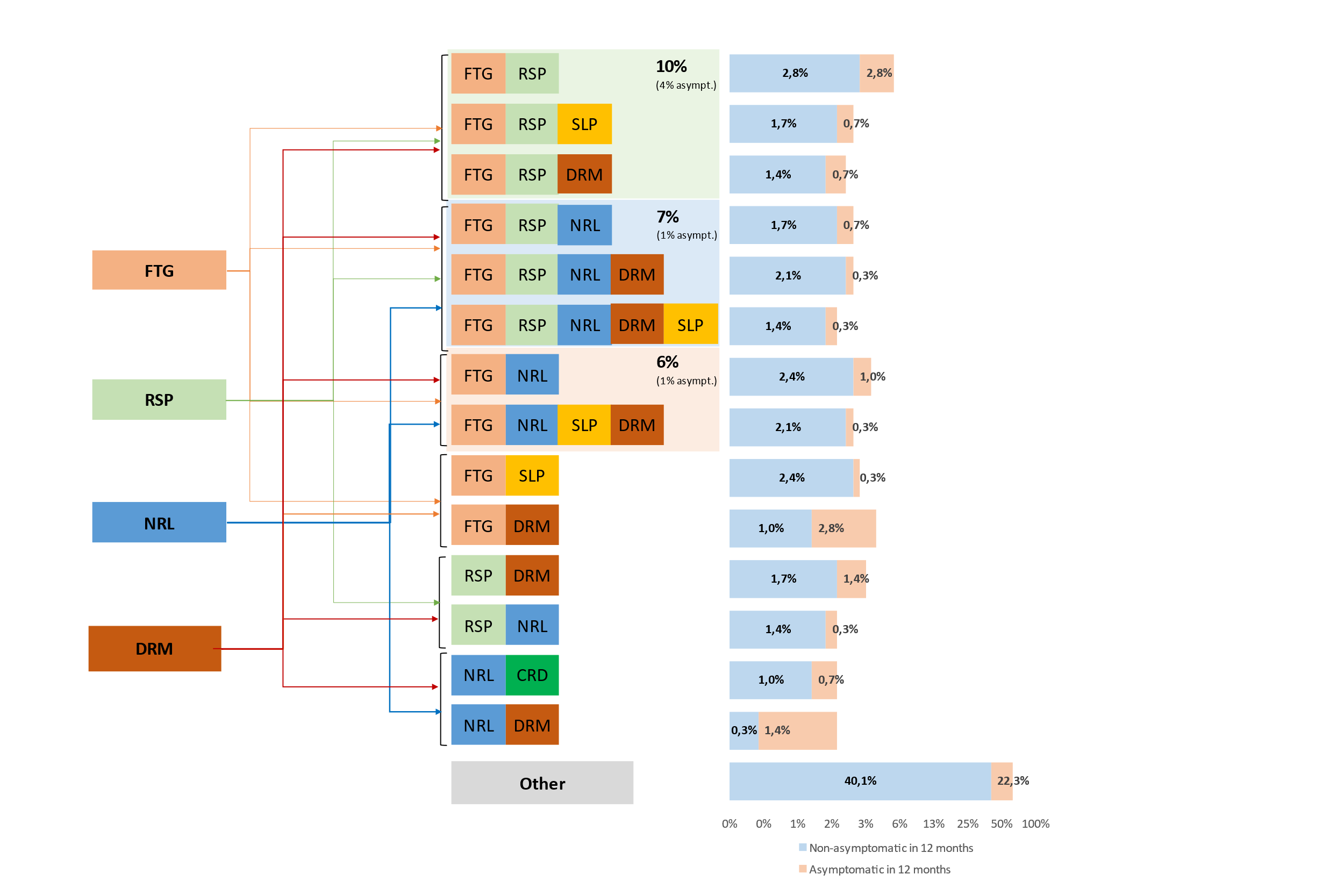

Supplement: Supplementary file 1 — Additional file 1: Table S1. Categorisation of symptoms. Table S2. Prevalence of post-COVID-19 condition manifestations in adults and children at first (6 months) and second (12 months) follow-up. Table S3. P values for prevalence of post-COVID-19 condition manifestations comparison between adults and children at first (6 months) and second (12 months) follow-up. Table S4. Characteristics (comorbidities and COVID-19 severity) among respondents and non-respondents (adults and children) at first (6 months) and second (12 months) follow-up. Figure S1. Post-COVID-19 condition phenotypes in adults at first (6 month) follow-up and subsequent complete resolving by 12 months. [file 12916_2022_2448_MOESM1_ESM.docx]
